# Cardiac Magnetic Resonance Imaging in the Diagnosis of Anterolateral Left Ventricular Ballooning, a Variant of Classic Takotsubo Cardiomyopathy

**DOI:** 10.1155/2012/537169

**Published:** 2012-11-12

**Authors:** R. Zbinden, M. Mutter, D. Weishaupt

**Affiliations:** ^1^Department of Cardiology, Triemlispital Zurich, 8063 Zurich, Switzerland; ^2^Department of Cardiology, Kantonsspital Glarus, 8750 Glarus, Switzerland; ^3^Department of Radiology, Triemlispital Zurich, 8063 Zurich, Switzerland

## Abstract

Transient left ventricular apical ballooning syndrome is characterized by transient akinesis of the left ventricular apex with basal wall hyperkinesis; this is also known as Takotsubo cardiomyopathy. There are three distinct contractile LV patterns described in the literature: apical, midventricular, and basal ballooning. The apical ballooning pattern is the most frequent pattern. We describe the case of a transient anterolateral left ventricular ballooning fulfilling the definition of Takotsubo cardiomyopathy except for the contractile LV pattern. The diagnosis was supported by cardiac magnetic resonance imaging and by the fact that the anterolateral ballooning resolved completely after 6 weeks.

## 1. Introduction

Transient left ventricular apical ballooning syndrome characterized by transient akinesis of the left ventricular apex with basal wall hyperkinesis was first described as Takotsubo-type cardiomyopathy in 1990 [1]. It was named for its distinctive appearance of the ventriculogram similar to the Japanese fishing instrument used for trapping octopuses, called Takotsubo. Takotsubo cardiomyopathy (TTC) is characterized by acute, profound, but reversible left ventricular (LV) dysfunction in the absence of significant coronary artery disease, triggered by acute emotional or physical stress. This phenomenon is identified by a distinctive pattern of “apical ballooning” and primarily affects postmenopausal women [2]. The majority of patients have a clinical presentation similar to that of acute coronary syndrome (ACS). TTC or stress-induced cardiomyopathy is a clinical syndrome which has been described only very recently and has been increasingly recognized outside Japan during the last few years. Diagnostic criteria are still vague; this is mainly due to the lack of knowledge about the etiology of TTC. Several mechanisms have been discussed such as multivessel epicardial spasm [3], catecholamine-induced cardiomyopathy [4], or microvascular dysfunction induced by mental stress [5].

We report the case of a stress-induced left ventricular anterolateral ballooning in a 61-year-old female patient matching the diagnostic criteria of TTC but with a distinct pattern of ballooning/dyskinesia. 

## 2. The Case

**Figure 1:**
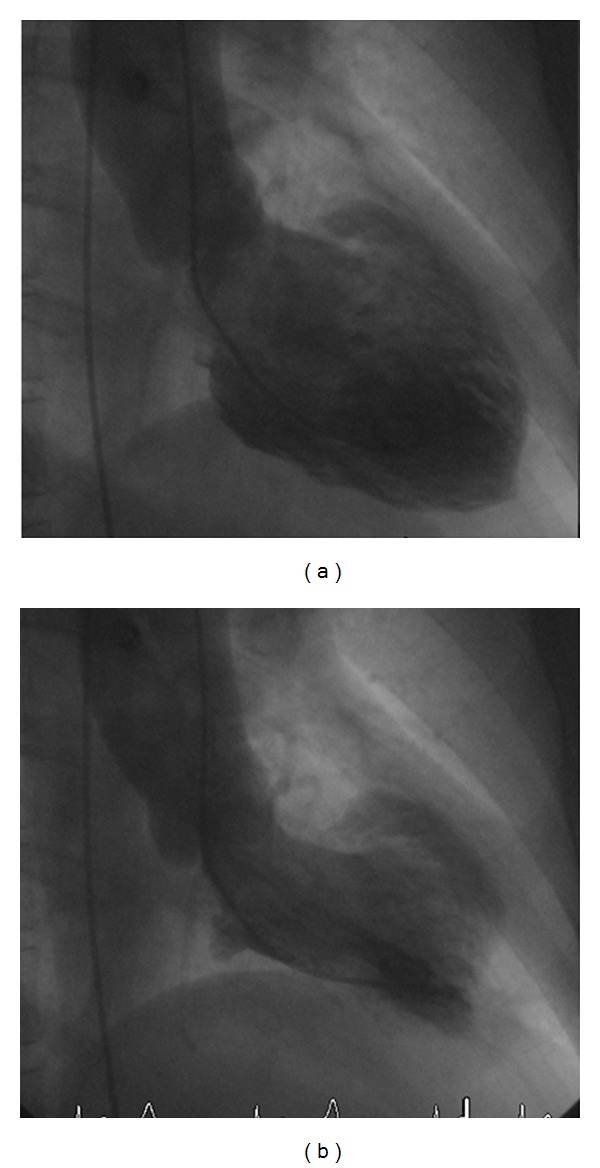
Left ventricular angiogram on admission revealing anterolateral ballooning.

**Figure 2:**
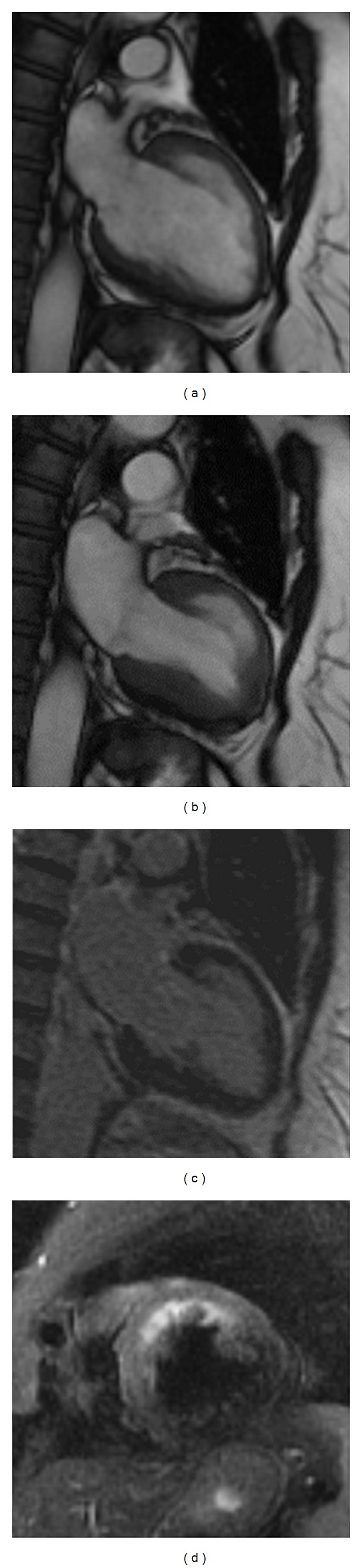
Cardiac magnetic resonance imaging. Enddiastolic and endsystolic 2-chamber view (upper row). Late enhancement 2-chamber view lower left. Anteroseptal edema in the T2 STIR short-axis view (lower right).

The 61 year old female patient presented the day after new years eve with typical anginal chest pain at rest with radiation to the jaw. The ECG at presentation showed minimal ST-elevation (0.5 mm) in I and aVL and poor R wave progression V1–V4. Regarding cardiovascular risk factors the patient had mild hypertension and dyslipidemia (currently not treated). Troponin test was positive with normal values vor CK/CK-MB. An emergency coronary angiography was performed which revealed normal coronary arteries. The left ventricular angiogram showed anterolateral ballooning with globally preserved ejection fraction (Figure 1). 24 h later a cardiac MRI was performed. We could confirm the anterolateral ballooning without any scar in that area (Figure 2). Additionally, signs of anterior edema were present on triple inversion recovery fast-spin echo sequence (T2 STIR). Asking the patient about a stressful event during the last few days, she admitted being very busy and stressed at home with a lot of relatives staying over new year's eve at her house. A pheochromocytoma as a cause for the transient ballooning has been excluded. 

The patient was diagnosed with an atypical form of takotsubo cardiomyopathy and discharged one day later with an ACE-inhibitor and a betablocker. 6 weeks later, the patient was seen as an outpatient. She was asymptomatic and LV function was completely normal on echo.

## 3. Discussion

Our case fulfills the current diagnostic criteria of stress-induced cardiomyopathy or takotsubo cardiomyopathy except for the contractile LV pattern [6]. In former studies, there were usually three distinct contractile LV patterns: apical, midventricular, and basal ballooning. In one large recent serie there was an additional biventricular (i.e., right and left ventricular) ballooning group. The apical ballooning pattern is the most frequent pattern. One similar case with focal anterolateral ballooning has been reported in the literature [7]. In this report the patient was female as well but older compared to our patient and the patient had nonsignificant coronary artery disease in the anterolateral region. Additionally, our patient underwent CMR imaging with characteristical findings of TTC (i.e., anterolateral edema, no scar on late enhancement imaging). 

Cardiovascular magnetic resonance (CMR) imaging is uniquely suited for the evaluation of patients with TTC. In addition to accurate visualization of regional wall motion abnormalities, it allows for precise quantification of right ventricular (RV) and LV function and the assessment of additional abnormalities (e.g., pericardial effusion, LV and RV thrombi, etc.). Importantly, CMR imaging also provides markers for reversible (inflammation, ischemic edema) and irreversible (necrosis/fibrosis) injury, which may be particularly important to verify TTC and exclude similar acute cardiac diseases such as myocardial infarction or myocarditis [8]. 

In conclusion we suggest that TTC can present not only with the classical LV ballooning patterns (i.e., apical, midventricular, basal) but also with segmental (i.e., anterolateral) ballooning, and CMR is a useful tool in the diagnosis of TTC.
